# Low coverage of hepatitis D virus testing in individuals with hepatitis B virus and HIV, the Netherlands, 2000 to 2022

**DOI:** 10.2807/1560-7917.ES.2025.30.7.2400344

**Published:** 2025-02-20

**Authors:** Anders Boyd, Colette Smit, Annemiek A van der Eijk, Hans Zaaijer, Bart JA Rijnders, Berend van Welzen, Mark AA Claassen, Katalin Pogány, Theodora EMS de Vries-Sluijs, Eline Op de Coul, Marc van der Valk, on behalf of the ATHENA observational cohort

**Affiliations:** 1 Stichting hiv monitoring, Amsterdam, the Netherlands; 2 Amsterdam UMC, location University of Amsterdam, Infectious Diseases, Amsterdam, the Netherlands; 3 Amsterdam Institute for Infection and Immunity, Infectious Diseases, Amsterdam, the Netherlands; 4 Department of Infectious Diseases, Public Health Service of Amsterdam, Amsterdam, the Netherlands; 5 Erasmus MC University Medical Center, Department of Viroscience, Rotterdam, the Netherlands; 6 Amsterdam UMC, location University of Amsterdam, Department of Clinical Virology, Meibergdreef 9, Amsterdam, the Netherlands; 7 Department of Internal Medicine, Section of Infectious Diseases and Department of Medical Microbiology and Infectious Diseases, Erasmus University Medical Center, Rotterdam, the Netherlands; 8 Department of Infectious Diseases, University Medical Centre Utrecht, Utrecht, the Netherlands; 9 Department of Internal Medicine and Infectious Diseases, Rijnstate Ziekenhuis, Arnhem, Netherlands; 10 Department of Internal Medicine, Maasstad Hospital, Rotterdam, the Netherlands; 11 Centre for Infectious Disease Control, Epidemiology and Surveillance, National Institute for Public Health and the Environment (RIVM), Bilthoven, the Netherlands; 12 Members of the ATHENA observational cohort are listed under Acknowledgements.

**Keywords:** Hepatitis B virus, hepatitis D virus, human immunodeficiency virus, hepatitis C virus, men who have sex with men, people who inject drugs, testing, public health, epidemiology, virus elimination

## Abstract

**Background:**

Since 2009, European guidelines recommend individuals with hepatitis B virus (HBV) and HIV be tested for hepatitis D virus (HDV).

**Aim:**

To analyse HDV testing in individuals with HBV/HIV during routine practice in the Netherlands.

**Methods:**

We assessed data from the ATHENA cohort of people with HIV who were ever HBV surface antigen-positive, aged ≥ 18 years and attended one of 24 HIV treatment centres in the Netherlands during 2000–22. Using longitudinal analysis, we estimated the percentage of individuals ever tested for HDV (antibody or RNA test) over time. In cross-sectional analysis, determinants for ever being tested by end of follow-up were assessed using relative risk regression.

**Results:**

We identified 1,715 individuals with HBV/HIV; 1,460 (85.1%) and 255 (14.9%) were male and female at birth, respectively (median age: 52 years; IQR: 42–59). Only 249 (14.5%) had an HDV test. The percentage tested increased from 5.0% (95% CI: 3.4–7.3) in 2000 to 17.0% (95% CI: 14.9–19.3) in 2022. In 2022, 16.2% (95% CI: 13.7–19.1) of men who have sex with men, 25.0% (95% CI: 9.7–50.9) of persons who inject(ed) drugs and 18.1% (95% CI: 14.6–22.3) of heterosexual/others were tested. In multivariable analysis, ever having an HDV test was associated with detectable HBV DNA viral load (p < 0.001), ever presenting with elevated alanine aminotransferase (ALT) levels (p = 0.023), advanced fibrosis/cirrhosis (p = 0.001) and being overweight/obese (p = 0.043).

**Conclusions:**

HDV testing coverage in the Netherlands is low for individuals with HBV/HIV. Although testing was more common in those with advanced liver disease, a considerable proportion at risk of HDV still need testing.

**Key public health message**

**What did you want to address in this study and why?**

Individuals with hepatitis B virus (HBV), which causes a chronic viral disease that can lead to liver cancer, are at substantial risk of hepatitis D virus infection (HDV). Together, these infections are associated with the highest risk of fatality among all hepatitis viruses. European guidelines are clear: everyone with HBV must be tested at least once for HDV. We assessed how common testing occurs in individuals with HBV and HIV in the Netherlands.

**What have we learnt from this study?**

As only 15% of individuals with HBV/HIV were tested for HDV, testing coverage is suboptimal. Although individuals with risk factors that would make them susceptible to hepatitis D virus, such as being a person who injects drugs, coming from a region of the world where hepatitis D virus is common or having severe liver damage were more frequently tested, there were still many individuals with HBV/HIV at clear risk of hepatitis D virus who were not tested.

**What are the implications of your findings for public health?**

These findings illustrate a shortcoming in the health services geared towards individuals with HBV/HIV, which could be apparent in other European settings. Clinicians need to be aware that individuals with HBV/HIV require testing so they can benefit from reductions in further transmission of hepatitis D virus, a more appropriate clinical follow-up and be considered for new effective pharmacological agents.

## Introduction

Hepatitis D virus (HDV), a satellite virus that requires hepatitis B virus (HBV) co-infection for replication, is associated with the highest risk of fatality among all hepatitis viruses [1]. Approximately 12 million people worldwide and 450,000 people in Europe have past or current HDV infection [2]. Given the severity of disease, Dutch and European guidelines have recommended since 2009 that all individuals with hepatitis B surface antigen (HBsAg)-positive serology be tested at least once for anti-HDV antibodies. In many countries in Europe, anti-HDV antibodies are more commonly found in people who inject(ed) drugs (PWID), individuals from high HDV endemic regions (i.e. anti-HDV antibody positive prevalence of > 5% in individuals with HBV, such as in parts of Central Asia, South America and Western Africa [2]) and men who have sex with men (MSM) [3]. HDV infection is typically treated with pegylated interferon-α, but rates of virological response, reflected by an undetectable HDV RNA, after treatment are expected to be less than 20%. Newer anti-HDV agents, such as bulevirtide, which leads to increased viral suppression and perhaps even virological response after treatment, have been included in clinical practice since 2023 [4]. Individuals testing positive for HDV could benefit from evaluation for treatment with these novel anti-HDV agents [5].

In countries without a generalised epidemic of the human immunodeficiency virus (HIV), individuals with HBV and HIV infection are almost seven times more likely to have HDV than those with HBV alone [2]. Furthermore, individuals with HIV, HBV and HDV have a higher risk of hepatocellular carcinoma and liver-related death than those with HBV/HIV [6]. In the Netherlands, individuals with HIV are more likely to belong to key populations for HDV infection (i.e. PWID, MSM) [7]. Given the possibility of effectively treating HDV in individuals with HBV/HIV, there is clearly a need to screen for HDV in this population.

Nevertheless, studies have shown a highly variable frequency of HDV testing during routine care for individuals with HBV/HIV, including 11% of those in care in the United States Veterans Affairs system [8], 55% in the Swiss HIV Cohort Study [6] and 56–100% at single centre studies [9-11] ever having an HDV test. The lack of HDV testing in some settings could translate into a substantial group of individuals who are not aware of their infection [12,13]. Importantly, the study periods of these studies do not overlap and cannot be compared, hence it is unclear whether improvements have been made in testing coverage over time [3,7].

The underlying reasons why individuals with HBV/HIV are tested for HDV are not well known; at the same time, any group at risk of HDV infection could benefit from targeted screening. In this study, we leveraged data from the ATHENA cohort to assess the changes in HDV testing between 2000 and 2022 in all individuals with HBV/HIV in care in the Netherlands, both overall and by key populations including PWID and MSM. We also aimed to understand the determinants of those who ever received an HDV test and of early HDV testing through a risk factor analysis.

## Methods

### Study design and participants

We conducted a longitudinal analysis and cross-sectional secondary analysis using data from the ATHENA cohort. In brief, HIV care in the Netherlands is provided by 24 treatment centres. After an initial visit, individuals with HIV regularly seek care at these treatment centres every 6 months for follow-up or as deemed necessary by the treating physician. As an integral part of HIV care, the Stichting hiv monitoring (SHM) [14] is responsible for prospectively collecting demographic data, relevant HIV and treatment details and viral hepatitis data from people with HIV in the Netherlands receiving care in one of the treatment centres.

This data collection is known as the ATHENA cohort [15], which was initiated in 1998 and captures data from more than 98% of all patients with HIV who are in care in the Netherlands. Data collection is continuous, and the database of the ATHENA cohort is locked twice per year, i.e. a version of the data is fixed, meaning no changes are possible, and cleaned. This analysis includes data from 1 January 2000 to 31 December 2022 (database lock June 2023).

Since 2002, the ATHENA cohort is managed by SHM, the institution appointed by the Dutch Ministry of Health, Welfare and Sport for the monitoring of people with HIV in the Netherlands. People entering HIV care receive written information about participation in the ATHENA cohort and are informed by their treating physician on the purpose of data collection. Thereafter, they can consent verbally or elect to opt-out. 

For the present study, we included all individuals in the ATHENA cohort database who ever had an HBsAg-positive serological test result before cohort entry, at first follow-up visit or during a subsequent follow-up visit.

### Study variables and data collection

We defined individuals who were tested for HDV as those who had an anti-HDV antibody test (using a commercially available assay) or an HDV RNA test (using an in-house qualitative test or if available, a quantitative test [16]).

Included individuals were assessed at the first visit for age, height, sex at birth, country of birth (grouped into regions of origin, as listed in Supplementary Table S1) and HIV transmission route (i.e. MSM, PWID, heterosexual contact and other modes of transmission), which we used to define key populations. We considered any MSM who ever injected drugs as belonging to the MSM key population. We collected data at the first visit and during follow-up visits on the following: CD4^+^ T-cell count, HIV-1 RNA and HBV DNA viral loads, AIDS-defining illness, hepatitis B ‘e’ antigen (HBeAg) status, alanine transaminase (ALT) and aspartate transaminase (AST) levels, platelets, liver fibrosis measurements, anti-HCV antibodies, HCV RNA viral loads, weight, blood pressure, glucose and lipid levels. Date of initiation and discontinuation for each HIV, HBV and HDV medication were also collected at each follow-up visit. We also identified whether individuals were receiving tenofovir-containing antiretroviral therapy (ART).

We estimated the duration of HBV infection as time in years since the first known HBsAg-positive test result until 31 December of each calendar year (for longitudinal analysis) or last date of follow-up in the cohort (for cross-sectional analysis). We divided the year of first HBsAg-positive test into two categories: before 2009 or in/after 2009. This year was selected as European guidelines began recommending screening for HDV in individuals with HBV in 2009. 

Advanced liver fibrosis or cirrhosis was considered to be present if the AST to platelet ratio index (APRI) score was > 2.0, liver stiffness ≥ 9.5 kPa or if diagnosed by liver biopsy. HCV-positive status was defined as a positive HCV RNA test. Alcohol use was physician reported and categorised into current, former and never use. Body mass index (BMI) was calculated as weight in kilograms divided by the height in metres squared and was categorised into normal/underweight (< 25 kg/m^2^) or overweight/obese (≥ 25 kg/m^2^). Hypertension was defined as having a systolic or diastolic blood pressure ≥ 160 or ≥ 100 mmHg, respectively, (i.e. grade 2 or higher hypertension based on criteria from the European Society of Cardiology [17]), diagnosis of hypertension from a physician or being treated for hypertension. Insulin resistance was defined as having one or more of the following: a fasting glucose level ≥ 7.0 mmol/l, regular glucose level ≥ 11.1 mmol/l, HbA1c level > 48 mmol/mol, diagnosis of type 1 or 2 diabetes mellitus from a physician or being treated for diabetes. An elevated triglyceride level was defined as having a serum triacylglycerol level > 1.7 mmol/L. Low high-density lipoprotein (HDL) cholesterol was defined as having an HDL cholesterol level < 1.0 and < 1.3 mmol/L for men and women, respectively.

### Statistical analysis

The observation period began at the individual's first cohort visit after 1 January 2000 and continued until the last visit before loss to follow-up, moving abroad, death or 31 December 2022, whichever occurred first. For those individuals who tested HBsAg-positive after cohort inclusion, their follow-up also began at the first cohort visit after 1 January 2000 to reduce immortal time bias from delayed HBV testing.

We described the study population at cross-sectional time points occurring every 5 years from 1 January 2007 until 1 January 2022. Counts and percentages were used for categorical variables and median and interquartile range (IQR) for continuous variables.

We first examined the yearly percentage of included individuals who were ever tested for HDV across calendar years from 1 January 2000 to 31 December 2022, both overall and within key populations. Additionally, we examined the percentage of individuals ever having an HDV test who had a positive anti-HDV test across these same calendar years. We modelled these outcomes over time using a logistic regression model with calendar year as restricted cubic splines in three knots.

To assess determinants of ever being tested for HDV, we used data from the last follow-up visit of all included individuals. We modelled the probability of ever being tested for HDV across levels of determinants using a Poisson regression model with robust variance estimation [18]. This model was used to estimate the relative risk (RR) and its 95% confidence interval (CI) for each determinant. We then included covariates with a p < 0.2 in a full multivariable model. After assessing for collinearity and stability of parameter estimates, i.e. overinflated variance, we removed covariates with a p > 0.05 in backwards stepwise fashion to arrive at a final multivariable model.

To assess determinants of early testing for HDV (i.e. HDV testing occurring at or within 30 days of first HBsAg-positive serology), we used data from the visit at which HDV testing took place of included individuals tested for HDV. We modelled the probability of being tested early for HDV across levels of determinants using the Poisson regression model described above. A multivariable model was constructed in the same manner as the model for HDV testing as an outcome.

To explore the between-centre variation, we examined the percentage of included individuals tested for HDV by the end of follow-up, regardless of whether they were still in care. Since testing patterns across centres could be affected by age, key populations and geographic origin, we also adjusted the percentage tested for HDV by these variables using logistic regression.

Statistical analysis was done using STATA (v15.1, StataCorp) and significance was determined using a p value < 0.05.

### Sensitivity analysis

Individuals who lose HBsAg might not need screening for HDV, particularly those able to clear their acute HBV infection. We conducted sensitivity analyses in which (i) individuals were censored at HBsAg-loss when examining changes in percentage ever tested for HDV over calendar years and (ii) individuals who did not have HBsAg-positive serology at their last follow-up visit were excluded when examining risk-factors for ever being tested for HDV.

## Results

**Figure 1:**
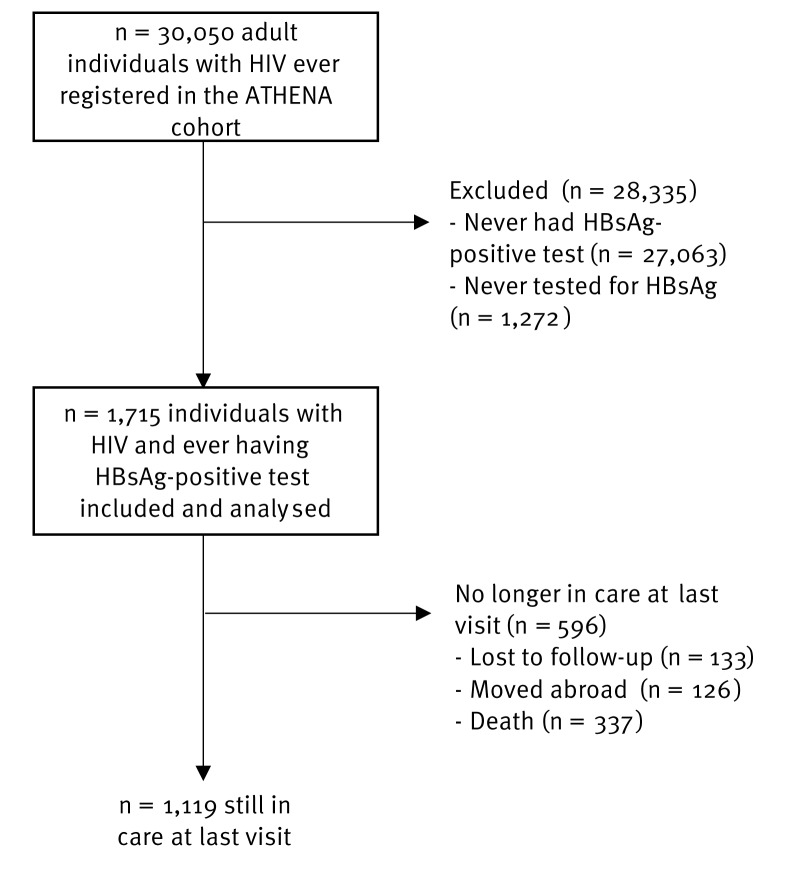
Flow chart of individuals included in the study from the ATHENA cohort, the Netherlands, 2000–2022

Up to 31 December 2022, there were 30,050 adult individuals with HIV ever registered in the ATHENA cohort. Of them, 1,715 had ever tested positive for HBsAg during follow-up and were included in the analysis (Figure 1). Median year of inclusion in the cohort was 2004 (IQR: 2002–11) and individuals were followed for a median 12.2 years (IQR: 4.7–18.5). Of those included, 1,460 (85.1%) and 255 (14.9%) were male and female at birth, respectively, and median age at the last visit was 52 (IQR: 42–59). 

HBsAg: hepatitis B surface antigen.

Table 1 describes individuals with HBV/HIV included in the longitudinal analysis according to specific 5-year periods from 2007 onwards. The median age of included individuals increased from 43 years in 2007 to 54 in 2022, while the percentage of male and female individuals remained similar during the same period. Most individuals in each 5-year period were MSM, which remained consistent over calendar years. Over time, the percentage of PWID as well as the percentage born in the Netherlands decreased. HIV RNA suppression also became more common and median CD4^+^ T-cell counts increased. Detectable HBV DNA was also uncommon during follow-up, but HBV DNA measurements were missing in the large majority of individuals (Table 1). The majority of individuals were HBeAg-negative as of their last measurement (987/1,559, 63.3%; missing data, n = 156). During follow-up, 468 (27.3%) of all included individuals (i.e. HBsAg-positive) became HBsAg-negative.

**Table 1:** Description of individuals with hepatitis B virus and HIV, the Netherlands, 2000–2022 (n = 1,715)

Characteristics	1 Jan 2007 (n = 881)		1 Jan 2012 (n = 1,042)		1 Jan 2017 (n = 1,080)		1 Jan 2022 (n = 1,095)	
	n	%	n	%	n	%	n	%
Age, median years (IQR)	43 (37–48)		47 (40–52)		51 (43–56)		54 (46–60)	
**Sex at birth**								
Male	761	86.4	905	86.9	941	87.1	941	85.9
Female	120	13.6	137	13.2	139	12.9	154	14.1
**HIV mode of transmission**								
MSM	557	63.2	658	63.2	700	64.8	698	63.7
Heterosexual	243	27.6	297	28.5	292	64.8	310	28.3
PWID	37	4.2	29	2.8	22	2.0	16	1.5
Other	44	5.0	58	5.6	66	6.1	71	6.5
**Region of origin**								
Netherlands	470	53.4	536	51.4	549	50.8	530	48.4
Western Europe	51	5.8	49	4.7	54	5.0	44	4.0
Sub-Saharan Africa	176	20.0	217	20.8	213	19.7	228	20.8
Caribbean/South America	97	11.0	126	12.1	131	12.1	141	12.9
South-east Asia	34	3.9	48	4.6	55	5.1	60	5.5
Other^a^	53	6.0	66	6.3	78	7.2	92	8.4
**HIV-related diagnosis**								
Ever AIDS	323	36.7	356	34.2	339	31.4	327	29.9
AIDS at HIV diagnosis	150	17.0	182	17.5	180	16.7	179	16.4
CD4^+^ T-cells/µL, median (IQR)^b^	459 (330–640)		550 (390–728)		610 (410–790)		640 (462–850)	
HIV RNA < 200 copies/mL^c^	589	68.7	816	79.5	966	90.5	961	91.0
**Current HIV treatment**								
Received combined ART	688	78.1	947	90.9	1047	96.9	1,093	99.8
Never received combined ART	193	21.9	95	9.1	36	3.4	1	0.1
Current tenofovir-containing ART	394	44.7	760	72.9	830	76.9	935	85.4
Ever received tenofovir-containing ART	451	51.2	854	82.0	994	92.0	1,062	97.0
**HBeAg status**								
Negative	442	50.2	564	54.1	611	56.6	660	60.3
Positive	327	37.1	369	35.4	361	33.4	330	30.1
Missing	112	12.7	109	10.5	108	10.0	105	9.6
**HBV DNA status**								
Detectable	157	17.8	129	12.4	91	8.4	70	6.4
Undetectable	146	16.6	174	16.7	148	13.7	164	15.0
Missing	578	65.6	739	70.9	841	77.9	861	78.6

AIDS: acquired immunodeficiency syndrome; ART: antiretroviral therapy; HBeAg: hepatitis B ‘e’ antigen; HBV: hepatitis B virus; HIV: human immunodeficiency virus; IQR: interquartile range; MSM: men who have sex with men; PWID: people who inject drugs.

^a^ Includes individuals from mostly North America, Eastern and Central Europe, Australia and the Asian-Pacific Region (outside South-east Asia).

^b^ Available data for CD4^+^ T-cell counts: 2007 (n = 842); 2012 (n = 933); 2017 (n = 866); 2022 (n = 760).

^c^ Available data for HIV RNA: 2007 (n = 857); 2011 (n= 1,026); 2017 (n = 1,067); 2022 (n = 1,056).

Data are from individuals with HBV/HIV. All characteristics refer to individuals in care at the date specified. Median calendar year of inclusion was 2004 (IQR: 2002–11).

Data source: ATHENA cohort.

### Hepatitis D virus testing in individuals with hepatitis B virus and HIV over time

**Figure 2:**
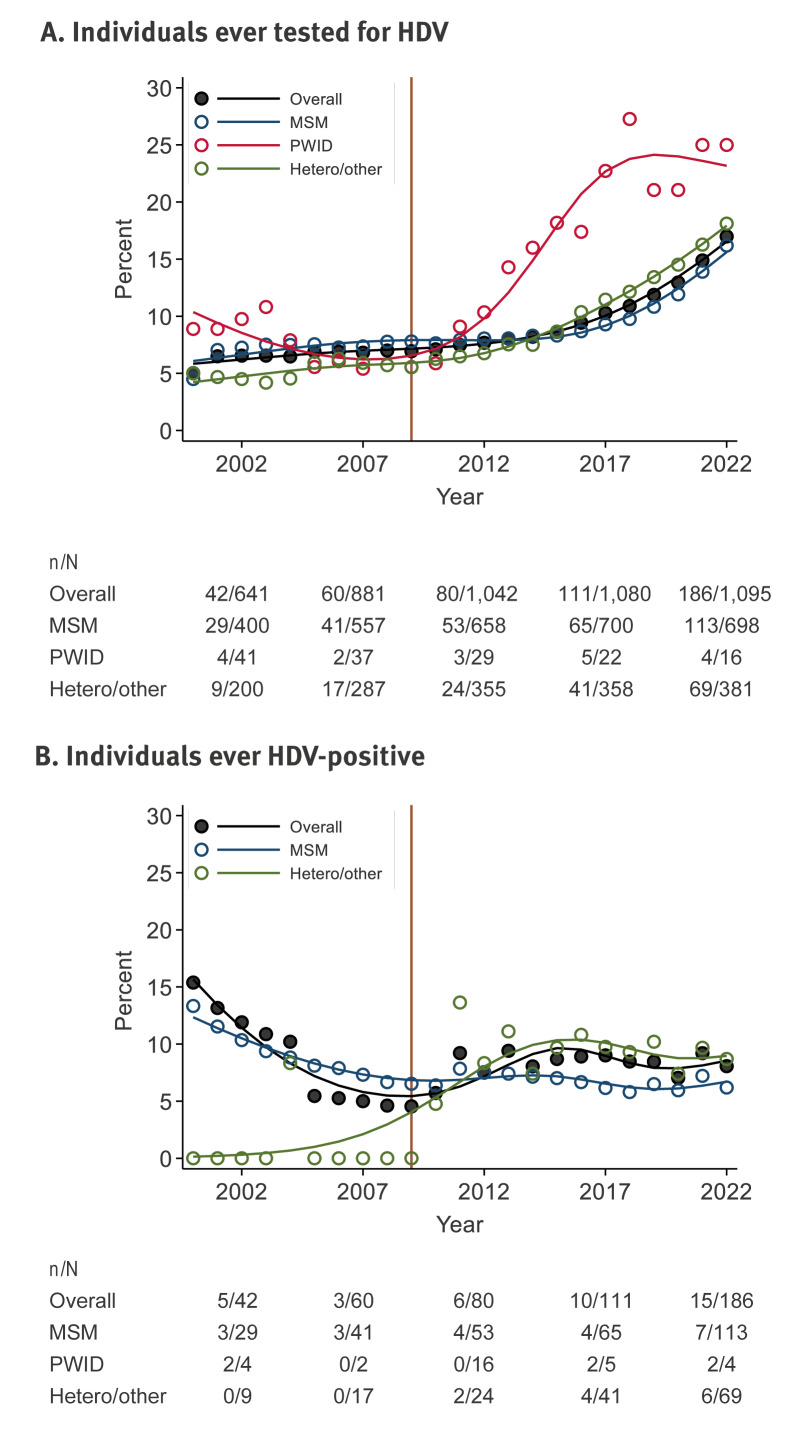
Hepatitis D virus testing and status in individuals with hepatitis B virus and HIV, the Netherlands, 2000–2022 (n = 1,715)

HIV: human immunodeficiency virus; Hetero/other: heterosexual or other mode of transmission; MSM: men who have sex with men; PWID: people who inject drugs.

Figure 2A shows the percentage of individuals with HBV/HIV ever receiving an HDV test per year. Of all those in care in 2000, 26 of 518 individuals (5.0%, 95% CI: 3.4–7.3) had ever received an HDV test; this increased to 186 of 1,095 individuals (17.0%, 95% CI: 14.9–19.3) by 2022. These increases were similarly observed in both the MSM (15/333, 4.5%, 95% CI: 2.7–7.3 in 2000 and 113/698, 16.2%, 95% CI: 13.6–19.1 in 2022) and heterosexual/other populations (7/140, 5.0%, 95% CI: 2.4–10.1 in 2000 and 69/381, 18.1%, 95% CI: 14.6–22.3 in 2022). Fluctuations were observed in PWID (4/45, 8.9%, 95% CI: 3.4–21.4 in 2000 and 4/16, 25.0%, 95% CI: 9.7–50.9 in 2022), likely owing to the small number of PWID remaining in care. In a sensitivity analysis, similar changes in the percentage ever tested for HDV, both overall and within key populations, was observed when censoring follow-up after HBsAg-loss. The percentage ever tested for HDV, both overall and within key populations, are summarised per year for this sensitivity analysis in Supplementary Figure S1.

The percentage of individuals tested for HDV is presented across calendar year in (A), stratified on key population. Of those tested for HDV, the percentage of individuals testing positive for HDV (i.e. anti-HDV positive or HDV RNA positive) is presented across calendar year in (B), stratified on key population. Circles represent the yearly percentages and lines are smoothed estimates of percentages from a logistic regression model with calendar year as restricted cubic splines in three knots. For every 5th calendar year starting from 2002 until 2022, the number with HDV testing of those in care (A) or with a positive HDV test among those tested (B) are provided at the bottom of the figure, for that specific calendar year. A vertical line at the year 2009 denotes the change in European guidelines that recommend HDV testing for all individuals with hepatitis B surface antigen positive serology.

Among those tested, the percentage of individuals with HBV/HIV who tested positive for HDV remained relatively stable over calendar years (Figure 2B). The number of individuals with positive HDV serology was 4 of 26 (15.4%, 95% CI: 5.9–34.6) in 2000 and 15 of 186 (8.1%, 95% CI: 4.9–12.9) in 2022. The percentage of individuals with positive HDV serology appeared to decrease in MSM, with large variation in yearly prevalence estimates (13.3%, 95% CI: 3.4–40.6 in 2000 and 6.2%, 95% CI: 3.0–12.4 in 2022), yet those in the heterosexual/other population had an increase from 0% in 2000 to 8.7% (95% CI: 4.0–18.1) in 2022. The few numbers of PWID ever tested for HDV across calendar years (range in n: 2–6) precluded analysis on the percentage with an HDV-positive result.

### Ever being tested for hepatitis D virus and its determinants in individuals with hepatitis B virus and HIV

By the last visit, 249 of 1,715 individuals (14.5%) had been tested for HDV. At the centre level, the percentage ever tested for HDV by the end of follow-up ranged from 0 to 28.3%, with rather wide variation between the 24 centres. This variation remained even after adjusting for age, key population and geographic origin. The distribution of centre-specific percentages of individuals with HBV/HIV who had an HDV test is provided in Supplementary Figure S2. Among those tested, 24 (9.6%) were positive for HDV; 13 of these individuals were tested for HDV RNA, of whom 11 (84.6%) had detectable HDV RNA.

The univariable associations between determinants and ever having an HDV test are given in Table 2. In a multivariable analysis (Table 2), ever having an HDV test was associated with being from Other regions (p = 0.004), ever receiving tenofovir-containing ART (p = 0.043), having a detectable HBV DNA viral load (p < 0.001), ever having ALT levels > 2 times the upper limit of normal (p = 0.023), having advanced fibrosis/cirrhosis (p = 0.001), being overweight or obese (p = 0.043) and having missing triglyceride levels during follow-up (p = 0.048). Of note, the percentage ever tested for HDV was no different between PWID and MSM (p = 0.64) or between those who were vs were not HCV-positive during follow-up (p = 0.30) in univariable analysis. In sensitivity analysis, the same factors in the main analysis were significant when excluding individuals who did not have HBsAg-positive serology at their last follow-up visit (the determinants of testing for hepatitis D virus in individuals included in this sensitivity analysis are provided in Supplementary Table S2), with the exception of ever receiving tenofovir-containing ART and missing information on triglyceride levels.

**Table 2:** Determinants of testing for hepatitis D virus in individuals with hepatitis B virus and HIV, the Netherlands, 2000–2022 (n = 1,715)

Characteristics	HDV testing				Univariable^a^			Multivariable^b^		
	No (n = 1,466)		Yes (n = 249)							
	n	%	n	%	RR	95% CI	p	aRR	95% CI	p
**Age, years**										
18–44	483	33.0	81	32.5	Ref.			NA		
45–55	464	31.6	79	31.7	1.02	0.77–1.35	0.88			
≥ 56	510	34.8	89	35.8	1.05	0.80–1.38	0.71			
Missing	9	0.6	0	0	NA					
**Sex at birth**										
Male	1,245	84.9	215	86.4	Ref.			NA		
Female	221	15.1	34	13.6	0.86	0.62–1.21	0.39			
**Region of origin**										
Netherlands	686	46.8	104	41.8	Ref.			Ref.		
Europe	85	5.8	10	4.0	0.92	0.50–1.69	0.79	0.86	0.45–1.65	0.64
Sub-Saharan Africa	329	22.4	60	24.1	1.15	0.86–1.54	0.34	1.16	0.85–1.56	0.35
Caribbean/South America	170	11.6	31	12.4	1.33	0.93–1.90	0.12	1.35	0.96–1.91	0.083
South-east Asia	68	4.6	11	4.4	1.11	0.64–1.92	0.72	1.14	0.62–2.11	0.68
Other	128	8.7	33	13.3	1.76	1.24–2.52	0.002	1.72	1.18–2.51	0.004
**Mode of transmission**										
MSM	845	57.6	140	56.2	Ref.			NA		
PWID	119	8.1	27	10.8	1.16	0.80–1.69	0.44			
Heterosexual/other	502	34.2	82	32.9	0.93	0.72–1.21	0.60			
**AIDS-defining illness**										
Never	943	64.3	170	68.3	Ref.			NA		
Ever	523	35.7	79	31.7	0.80	0.63–1.03	0.08			
**Year of HBV diagnosis**										
< 2009	1050	71.6	161	64.7	Ref.			NA		
≥ 2009	401	27.4	88	35.3	1.41	1.12–1.79	0.004			
Missing	15	1.0	0	0	NA					
**Tenofovir-containing ART**										
Never	236	16.1	11	4.4	Ref.			Ref.		
Ever	1,230	83.9	238	95.6	3.76	2.10–6.73	< 0.001	1.90	1.02–3.54	0.043
**Detectable HBV DNA viral load during follow-up**										
Never	657	44.8	36	14.5	Ref.			Ref.		
Ever	809	55.2	213	85.5	3.96	2.82–5.57	< 0.001	2.96	1.01–1.59	< 0.001
**HBeAg-positive during follow-up**										
Never	707	48.2	92	37.0	Ref.			NA		
Ever	759	51.8	157	63.0	1.54	1.23–1.94	< 0.001			
**ALT > 2x ULN**										
Never	891	72.6	143	61.4	Ref.			Ref.		
Ever	336	27.4	90	38.6	1.40	1.11–1.77	0.004	1.32	1.04–1.68	0.023
Missing	239	16.3	16	6.4	NA			NA		
**ALT > 5 ULN**										
Never	1,040	84.8	187	80.3	Ref.			NA		
Ever	187	15.2	46	19.7	1.21	0.91–1.62	0.19			
Missing	239	16.3	16	6.4	NA					
**Advanced fibrosis/cirrhosis**										
Absent	1,104	75.3	187	75.1	Ref.			Ref.		
Present	162	11.1	57	22.9	1.67	1.30–2.15	< 0.001	1.55	1.18–2.02	0.001
Missing	200	13.6	5	2.0	0.15	0.06–0.37	< 0.001	NA		
**HCV-RNA-positive during follow-up**										
Never	1,353	92.3	234	94.0	Ref.			NA		
Ever	113	7.7	15	6.0	0.74	0.46–1.21	0.23			
**Alcohol use**										
Never	485	33.1	59	23.7	Ref.			NA		
Former	260	17.7	43	17.3	1.62	1.10–2.37	0.25			
Current	721	49.2	147	59.0	1.55	1.13–2.11	0.001			
**BMI category**										
Normal/underweight	780	53.2	114	45.8	Ref.			Ref.		
Overweight/obese	566	38.6	118	47.4	1.30	1.03–1.64	0.026	1.26	1.01–1.59	0.043
Missing	120	8.2	17	6.8	NA			NA		
**Hypertension during follow-up**										
Never	808	55.1	131	52.6	Ref.			NA		
Ever	601	41.0	117	47.0	1.15	0.92–1.44	0.21			
Missing	57	3.9	1	0.4	0.12	0.02–0.84	0.033			
**Insulin resistance during follow-up**										
Never	1,107	75.5	197	79.1	Ref.			NA		
Ever	249	17.0	46	18.5	1.08	0.81–1.45	0.59			
Missing	110	7.5	6	2.4	0.34	0.16–0.76	0.009			
**Elevated triglycerides during follow-up**										
Never	310	21.2	75	30.1	Ref.			Ref.		
Ever	1,044	71.2	165	66.3	0.74	0.58–0.95	0.071	0.81	0.62–1.06	0.13
Missing	112	7.6	9	3.6	0.34	0.18–0.65	0.001	0.46	0.22–0.99	0.048
**Low HDL cholesterol during follow-up**										
Never	448	30.6	93	37.4	Ref.			NA		
Ever	825	56.3	137	55.0	0.91	0.72–1.16	0.46			
Missing	193	13.2	19	7.6	0.44	0.28–0.70	0.001			

AIDS: acquired immunodeficiency syndrome; ALT: alanine aminotransferase; APRI: aspartate aminotransferase to platelet ratio index; aRR: adjusted relative risk; BMI: body mass index; CI: confidence interval; HBeAg: hepatitis B ‘e’ antigen; HBV: hepatitis B virus; HCV: hepatitis C virus; HDV: hepatitis D virus; HDL: high density lipoprotein; NA: not applicable i.e. variable or category level was not considered in the model; RR: relative risk; ULN: upper limit of normal.

^a^ All parameter estimates were adjusted by clinical site.

^b^ In the multivariable model, HBeAg status and year of HBV diagnosis before or in/after 2009 were excluded as they were collinear with all other HBV-related markers. ALT > 2x ULN was more frequent and was chosen over ALT > 5x ULN for more stable estimates. The parameter estimates for hypertension were unstable, hence excluded from the model. The remaining candidate variables (i.e. AIDS-defining illness, alcohol use, low HDL cholesterol during follow-up, and insulin resistance) were excluded as their p-value was > 0.05. All parameter estimates were additionally adjusted by clinical site.

Data are from individuals with HBV/HIV, including information from cohort inclusion until the last clinical visit between 2000 and 2022. All characteristics refer to the date of last clinical visit unless otherwise specified.

Data source: ATHENA cohort.

### Early testing for hepatitis D virus and determinants in individuals with hepatitis B virus and HIV 

Of the 249 individuals tested for HDV, 62 (24.9%) were tested early (i.e. within 30 days of HBsAg-positive result). The remaining 187 individuals were tested for HDV a median 5.3 years (IQR: 1.2–13.8) after their first HBsAg-positive test. Of note, HDV testing also occurred at or within 30 days of coming into care for HIV (n = 51), or a median 9.2 years (IQR: 2.9–16.5) after HIV diagnosis (n = 169).

The univariable associations between determinants and early HDV testing (i.e. testing occurring within 30 days after first HBsAg-positive test) are given in Table 3. In a multivariable analysis (Table 3), early testing for HDV was associated with being from western Europe or Other region of origin (p = 0.006 and 0.019, respectively) and being diagnosed with HBV after 2009 (p < 0.001).

**Table 3:** Determinants of early testing for hepatitis D virus in individuals with hepatitis B virus and HIV who had been tested for HDV, the Netherlands, 2000–2022 (n = 249)

Determinant at HDV test	Early HDV testing				Univariable^a^			Multivariable^b^		
	No (n = 187)		Yes (n = 62)							
	n	%	n	%	RR	95% CI	p	RR	95% CI	p
**Age, years**										
18–44	61	32.6	20	32.3	Ref.			NA		
45–55	54	28.9	25	40.3	1.63	0.95–2.79	0.078			
≥ 56	72	38.5	17	27.4	0.91	0.50–1.68	0.77			
**Sex at birth**										
Male	162	86.6	53	85.5	Ref.			NA		
Female	25	13.4	9	14.5	1.06	0.58–1.96	0.85			
**Region of origin**										
Netherlands	82	43.8	22	35.5	Ref.			Ref.		
Europe	5	2.7	5	8.1	2.84	1.36–5.94	0.005	3.15	1.38–7.21	0.006
Sub-Saharan Africa	46	24.6	14	22.6	1.00	0.54–1.86	0.99	1.05	0.56–1.96	0.88
Caribbean/South America	27	14.4	4	6.4	0.59	0.20–1.72	0.34	0.55	0.18–1.63	0.28
South-east Asia	8	4.3	3	4.8	1.29	0.41–4.04	0.66	0.92	0.32–2.68	0.88
Other	19	10.2	14	22.6	2.28	1.28–4.06	0.005	1.82	1.11–2.99	0.019
**Mode of transmission**										
MSM	114	61.0	26	41.9	Ref.			NA		
PWID	14	7.5	13	21.0	2.45	1.36–4.41	0.003			
Heterosexual/other	59	31.6	23	37.1	1.42	0.86–2.33	0.17			
**AIDS-defining illness**										
No	130	69.5	40	64.5	Ref.			NA		
Yes	57	30.5	22	35.5	1.23	0.81–1.88	0.34			
**Year of HBV diagnosis**										
< 2009	136	72.7	25	40.3	Ref.			Ref.		
≥ 2009	51	27.3	37	59.7	2.80	1.73–4.53	<0.001	2.49	1.55–4.00	<0.001
**Tenofovir-containing ART**										
No	11	5.9	13	21.0	Ref.			NA		
Yes	176	94.1	49	79.0	0.39	0.23–0.65	<0.001			
**Detectable HBV DNA viral load**										
No	25	13.4	11	17.7	Ref.			NA		
Yes	162	86.6	51	82.3	0.81	0.45–1.45	0.48			
**HBeAg-positive**										
No	67	35.8	25	40.3	Ref.			NA		
Yes	120	64.2	37	59.7	1.00	0.64–1.55	0.99			
**ALT > 2x ULN**										
No	95	50.8	30	48.4	Ref.			NA		
Yes	54	28.9	28	45.2	1.62	1.05–2.52	0.031			
Missing	38	20.3	4	6.4	NA					
**ALT > 5 ULN**										
No	121	64.7	41	66.1	Ref.			NA		
Yes	28	15.0	17	27.4	1.70	1.06–2.73	0.028			
Missing	38	20.3	4	6.5	NA					
**Advanced fibrosis/cirrhosis**										
Absent	131	70.0	40	64.5	Ref.			NA		
Present	34	18.2	21	33.9	1.61	1.03–2.52	0.037			
Missing	22	11.8	1	1.6	NA					
**HCV-RNA-positive**										
No	174	93.1	60	96.8	Ref.			NA		
Yes	13	7.0	2	3.2	0.54	0.15–1.93	0.35			
**Alcohol use**										
None	74	39.6	19	30.7	Ref.			NA		
Former	29	15.5	8	12.9	0.97	0.46–2.05	0.95			
Current	84	44.9	35	56.4	1.21	0.72–2.01	0.47			
**BMI category**										
Normal/underweight	77	41.2	19	30.6	Ref.			NA		
Overweight/obese	66	35.3	6	9.7	0.54	0.22–1.35	0.19			
Missing	44	23.5	37	59.7	NA					
**Hypertension**										
No	99	52.9	31	50.0	Ref.			NA		
Yes	86	46.0	31	50.0	1.17	0.74–1.85	0.49			
Missing	2	1.1	0	0	NA					
**Insulin resistance**										
No	149	79.7	47	75.8	Ref.			NA		
Yes	34	18.2	12	19.4	1.12	0.64–1.93	0.69			
Missing	4	2.1	3	4.8	2.00	0.71–5.67	0.19			
**Elevated triglycerides**										
No	48	25.7	27	43.6	Ref.			NA		
Yes	133	71.1	31	50.0	0.54	0.34–0.85	0.008			
Missing	6	3.2	4	6.4	1.41	0.55–3.59	0.47			
**Low HDL cholesterol**										
No	65	34.8	28	45.2	Ref.			NA		
Yes	110	58.8	26	41.9	0.67	0.41–1.09	0.11			
Missing	12	6.4	8	12.9	1.53	0.75–3.13	0.25			

AIDS: acquired immunodeficiency syndrome; ALT: alanine aminotransferase; APRI: aspartate aminotransferase to platelet ratio index; aRR: adjusted relative risk; BMI: body mass index; CI: confidence interval; HBeAg: hepatitis B ‘e’ antigen; HBV: hepatitis B virus; HCV: hepatitis C virus; HDV: hepatitis D virus; HDL: high density lipoprotein; NA: not applicable i.e., variable or category level was not considered in the model; RR: relative risk; ULN: upper limit of normal.

^a^ All parameter estimates were adjusted by clinical site.

^b^ In the multivariable model, age and mode of transmission were excluded because of collinearity with region of origin, tenofovir-containing ART because of collinearity with diagnosis before or in/after 2009, and ALT levels > 2 or 5 ULN because of collinearity with advanced fibrosis/cirrhosis. The remaining candidate variables (i.e. overweight/obese, elevated triglycerides, low HDL cholesterol, advanced fibrosis/cirrhosis) were excluded as their p-value was > 0.05. All parameter estimates were additionally adjusted by clinical site.

Data are from individuals with HBV/HIV, including information from cohort inclusion until the last clinical visit from 2000 to 2022. All characteristics refer to the date of HDV testing unless otherwise specified.

Data source: ATHENA cohort.

## Discussion

In the Netherlands, roughly 15% of individuals with HBV/HIV have been tested for HDV based on data from a nationwide cohort. The proportion of individuals currently in care who have been ever tested for HDV only slightly increased over the past 2 decades, from roughly 5% in 2000 to 17% in 2022. This increase coincided after European recommendations to screen all individuals with HBsAg-positive serology for HDV. These data highlight the poor coverage of HDV testing for individuals who are clearly recommended to be screened at least once for HDV [4]. The lack of HDV testing limits our understanding of the HDV epidemic and more broadly, how much progress has been made to achieve the actions laid out in the recent Global health sector strategies on viral hepatitis from the World Health Organization [19].

Globally, HDV testing coverage is extremely variable and depends on several factors. At the structural level, receiving care at a primary healthcare centre, as opposed to a hepatology centre or academic hospital, is associated with much lower HDV testing coverage [20,21]. Centres that have incorporated automatic testing of anti-HDV antibodies after an HBsAg-positive result (i.e. double reflex testing) have seen HDV testing coverage above 93% [20,22,23], although double reflex testing requires programmatic changes that might be difficult for some healthcare structures to implement. Still, it remains one of the missing components to more accurately estimate the number of HDV infections worldwide [24]. The wide variation in HDV testing coverage across sites in our study could be a reflection of these structural level influences – a handful of centres in this study were academic, where double reflex testing has been implemented within the past 3 years. At an individual level, studies in large networks of clinical centres in the United States have shown that certain ethnic groups (i.e. Black, Asian) are more likely to receive testing than Caucasians [25,26]. Importantly, individuals with HBV/HIV are less often tested for HDV than those with only HBV [21,27], thereby stressing the need for HDV testing in those with HBV and HIV.

In Europe, several groups of individuals are at higher risk of having anti-HDV antibodies, including PWID and those from HDV-endemic regions [28,29]. It would be expected that these individuals are also more frequently screened for HDV. In fact, we observed no difference in the proportion tested for HDV between key populations, nor a difference between individuals from sub-Saharan Africa than those from the Netherlands. The lack of difference between these key populations is probably explained by the very few PWID included in our cohort, which is the result of the higher mortality observed in this population before the ATHENA cohort had been initiated and the fact that PWID in the Netherlands, particularly with HCV or HIV, might be underserviced and not actively engaged in care [30,31].

More frequent HDV testing did, however, occur in individuals with more active forms of HBV infection (i.e. ever having detectable HBV DNA, HBeAg-positive serology). Considering that active HDV infection generally suppresses HBV DNA replication and is more commonly found in HBeAg-negative individuals [32-34], HDV testing in those with detectable HBV DNA or HBeAg-positive serology might not be an ideal strategy for targeted screening. However, this finding more likely reflects the routine work-up involved with elevated transaminase levels [4], rather than intent to screen for HDV. Early testing (i.e. ≤ 30 days after HBsAg-positive result) was more frequent when European guidelines started recommending systematic HDV testing (i.e. 2009 onwards) and in individuals with advanced liver fibrosis/cirrhosis, both of which could indicate more protocolled approaches to HDV testing.

Indeed, individuals with elevated ALT levels (i.e. twice the ULN) were also more frequently tested for HDV. Routine evaluation of abnormal liver enzymes does include additional assessments of metabolic syndrome, liver steatosis and intake of hepatotoxic drugs [35]. It could be hypothesised that if other causes of elevated liver enzymes are identified during early routine work-up, testing for HDV might be ignored. In this instance, we would expect much lower proportions with an HDV in individuals with signs of metabolic syndrome or steatosis. We did not observe a difference in HDV testing between individuals with and without hypertension, insulin resistance, elevated triglycerides and lower HDL, while HDV testing was more frequent in overweight or obese individuals. Our data would suggest that HDV testing is not being limited by other aetiologies of liver disease.

HDV results in an accelerated progression of liver fibrosis and hepatocellular carcinoma [36], particularly for individuals with HIV [6]. The more frequent HDV screening in individuals with advanced liver fibrosis or cirrhosis likely reflects the need to identify its underlying aetiology. Although this finding is encouraging, 83% of the individuals in our cohort with advanced liver fibrosis or cirrhosis were never tested for HDV [37].

An often-used criterion for appropriate screening is ‘there should be an accepted treatment for patients with recognised disease’ [38]. The current therapeutic option worldwide for HDV is standard or pegylated interferon-α with anti-HBV treatment [4,39], which has limited efficacy and low tolerability in individuals with HIV [40]. In addition, currently available anti-HBV agents, namely tenofovir, bear little effect on HDV replication over time [41,42] and do not produce rates of viral clearance that are any higher than during the natural course of HDV infection [43]. It is then debatable whether HDV testing should be expanded in the current context. Nevertheless, the novel agent bulevirtide has demonstrated high rates of viral HDV clearance in early compassionate use [44,45] and it has been used in individuals with HIV [46]. Other anti-HDV agents that have shown evidence of potential clinical efficacy are in development [47]. Identifying individuals with HDV, specifically with active HDV RNA replication, could assist in estimating the need for novel therapeutic agents in the Netherlands.

Our study has several limitations. Firstly, we determined HDV testing status directly from laboratory records at each centre. We are unable to account for testing that may have taken place internally without referral to the hospital laboratory or HDV testing that might have occurred at other locations. Furthermore, we did not ask whether individuals had been tested for HDV before inclusion in the ATHENA cohort. These biases could have resulted in an underestimation of HDV testing. Secondly, we rely on the thoroughness of routine data collection during clinical consultations. Some data, such as behaviours associated with HBV/HDV infection and migration history for those who were born in the Netherlands but their parents were not, are not routinely collected and hence could not be used in analysis. We also did not have data on the most likely transmission route for HBV/HDV infection and MSM who engaged in injecting drug use were considered as MSM and not PWID. Inference on HDV testing in these key population needs to be considering in light of this potential for misclassification. Finally, individuals with isolated anti-HBcAb positive may also have active HBV replication and indeed, 4% of those with this serological profile and HIV are estimated to have detectable HBV DNA [48]. HDV testing could also be advised for these individuals. Yet considering that we did not have data on HBV DNA for individuals with isolated anti-HBcAb-positive serology, it could not be assessed.

## Conclusions

The coverage of HDV testing, at 14%, is low in individuals with HBV and HIV in the Netherlands. HIV specialists should be aware of the risk of HDV infection for their patients with HBV/HIV and should strive to increase HDV testing coverage. For most of the individuals tested, delays of a median 5 years after HBsAg positive serology occurred, highlighting the need for improvements in anti-HDV antibody and HDV RNA testing. Such improvements could be made through double reflex testing when HBsAg and anti-HDV antibody status are positive, respectively. A large proportion of individuals at risk for HDV still need to be tested for HDV, while the true number of individuals with HBV and HIV who have active HDV replication is likely higher than what is reported in our study. Expanded HDV testing is needed to confirm this claim.

## Supplementary Data

189000 Supplement
