# Supplementary material for: Low coverage of hepatitis D virus testing in individuals with hepatitis B virus and HIV, the Netherlands, 2000 to 2022
Source: Euro Surveill. 2025 Feb 20;30(7):2400344. doi: 10.2807/1560-7917.ES.2025.30.7.2400344 (PMC11843617; doi:10.2807/1560-7917.ES.2025.30.7.2400344)
Supplement: Supplement [file 24-00344_BOYD_Supplement.pdf]

Supplement to: Low coverage of hepatitis D virus testing in individuals with hepatitis B virus and HIV, the Netherlands, 2000 to 2022

Table of contents

SUPPLEMENTARY FIGURES 2
Supplementary Figure S1. Hepatitis D virus testing in individuals with hepatitis B virus and HIV in the Netherlands (among those with positive hepatitis B surface antigen during follow-up) 2
Supplementary Figure S2. Distribution of centre-specific percentages of individuals with HBV and HIV who had an HDV test 3
SUPPLEMENTARY TABLES 4
Supplementary Table S1. Countries included for each region of origin used in analysis 4
Supplementary Table S2. Determinants of testing for hepatitis D virus in individuals with HBV/HIV in the Netherlands (among those with positive hepatitis B surface antigen at their last follow-up visit) 5

This supplementary material is hosted by *Eurosurveillance* as supporting information alongside the article ‘Low coverage of hepatitis D virus testing in individuals with hepatitis B virus and HIV infection, the Netherlands, 2000 to 2022’, on behalf of the authors, who remain responsible for the accuracy and appropriateness of the content. The same standards for ethics, copyright, attributions and permissions as for the article apply. Supplements are not edited by *Eurosurveillance* and the journal is not responsible for the maintenance of any links or email addresses provided therein.

SUPPLEMENTARY FIGURES

Supplementary Figure S1. Hepatitis D virus testing in individuals with hepatitis B virus and HIV in the Netherlands (among those with positive hepatitis B surface antigen during follow-up)

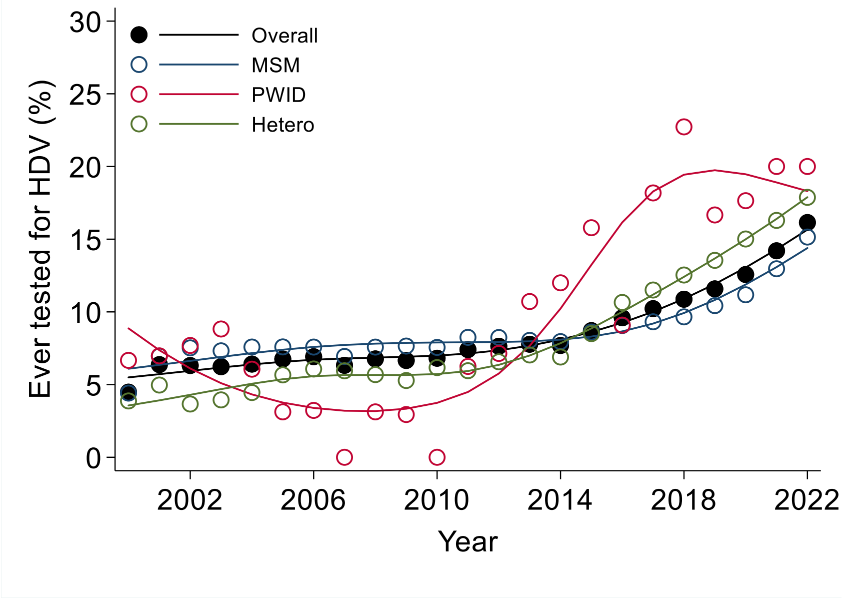

N						
Overall	602	766	910	1014	1030	1053
MSM	372	488	569	641	673	680
PWID	39	31	28	25	22	15
Hetero	191	247	299	335	335	358

Key populations included men who have sex with men (MSM), people who inject drugs (PWID) and those who acquired HIV through heterosexual transmission (hetero). The lattermost group also includes individuals from all other transmission categories. The percentage of individuals tested for hepatitis D virus (HDV) is presented across calendar year, stratified on key population.

Supplementary Figure S2. Distribution of centre-specific percentages of individuals with HBV and HIV who had an HDV test

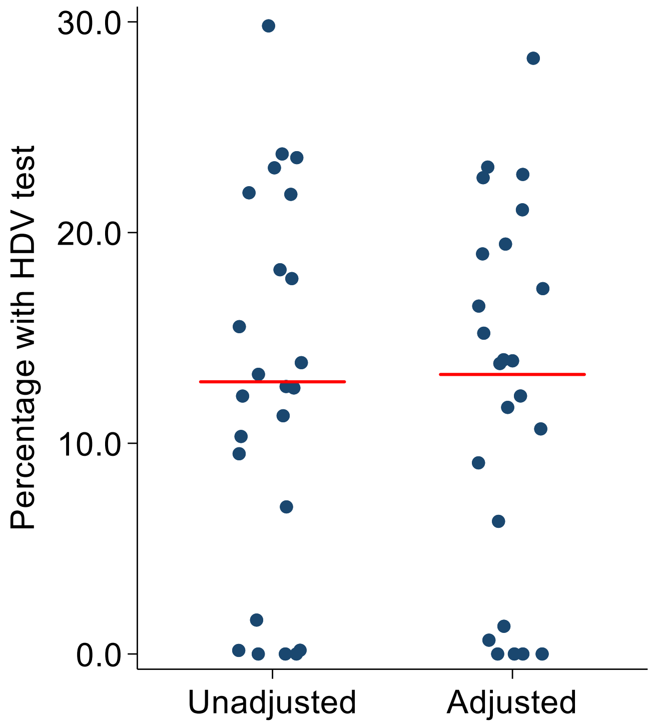

Individual dots represent center-level percentage of individuals with HBV and HIV who had been tested for HDV by the end of follow-up, regardless of whether they were still in care. This percentage is provided both unadjusted and adjusted for age, key population, and geographic origin. The red line depicts the median level across centers.
Abbreviations: HBV, hepatitis B virus; HDV, hepatitis D virus; HIV, human immunodeficiency virus.

SUPPLEMENTARY TABLES

Supplementary Table S1. Countries included for each region of origin used in analysis

Western Europe	Sub-Saharan Africa	Carribbean/South America	South-east Asia	Other
Austria	Angola	Argentina	Cambodia	Australia
Belgium	Benin	Aruba	China	Bosnia and Herzegovina
Finland	Burkina Faso	Bahamas	India	Bulgaria
France	Burundi	Bolivia	Indonesia	Canada
Germany	Cabo Verde	Brazil	Iran	Estonia
Greece	Cameroon	Chile	Lao People's Democratic Republic	Iraq
Ireland	Central African Republic	Colombia	Malaysia	Japan
Italy	Chad	Cuba	Myanmar	South Korea
Netherlands	Congo (the Democratic Republic of the)	Curaçao	Nepal	Latvia
Norway	Congo	Dominican Republic	Philippines	Lebanon
Portugal	Côte d'Ivoire	Dutch Antillies	Sri Lanka	Lithuania
Spain	Eritrea	Ecuador	Thailand	Morocco
Switzerland	Ethiopia	French Guiana	Viet Nam	Papua New Guinea
United Kingdom of Great Britain and Northern Ireland	Gambia	Jamaica		Poland
	Ghana	Mexico		Romania
	Guinea	Panama		Russian Federation
	Guinea-Bissau	Peru		Slovakia
	Kenya	Suriname		Sudan
	Liberia	Trinidad and Tobago		Syrian Arab Republic
	Mali	Venezuela (Bolivarian Republic of)		Turkey
	Mauritania			Ukraine
	Mozambique			United States of America
	Namibia			
	Niger			
	Nigeria			
	Rwanda			
	Senegal			
	Sierra Leone			
	Somalia			
	South Africa			
	Tanzania, United Republic of			
	Togo			
	Uganda			
	Zambia			
	Zimbabwe			

Supplementary Table S2. Determinants of testing for hepatitis D virus in individuals with HBV/HIV in the Netherlands (among those with positive hepatitis B surface antigen at their last follow-up visit)

		Univariable€		Multivariable¥	
		RR (95%CI)	*p*	aRR (95%CI)	*p*
Region of origin					
	the Netherlands	Ref		Ref	
	Europe	0.57 (0.26-1.27)	0.17	0.50 (0.20-1.26)	0.14
	Sub-Saharan Africa	1.01 (0.75-1.36)	0.97	1.13 (0.81-1.56)	0.47
	Caribbean/South America	1.19 (0.82-1.73)	0.36	1.23 (0.85-1.78)	0.26
	Southeast Asia	0.88 (0.49-1.57)	0.67	0.98 (0.52-1.86)	0.95
	Other	1.51 (1.02-2.22)	0.037	1.69 (1.12-2.54)	0.012
Tenofovir-containing ART					
	Never	Ref		Ref	
	Ever	3.68 (2.01-6.75)	<0.001	1.82 (0.96-3.46)	0.067
Detectable HBV DNA viral load during follow-up					
	Never†	Ref		Ref	
	Ever	3.32 (2.21-4.99)	<0.001	2.19 (1.36-3.52)	0.001
ALT >2x ULN					
	Never†	Ref		Ref	
	Ever	1.50 (1.17-1.92)	0.001	1.40(1.08-1.82)	0.012
Advanced fibrosis/cirrhosis‡					
	Absent	Ref		Ref	
	Present	1.60 (1.23-2.08)	<0.001	1.52 (1.14-2.02)	0.005
	Missing	0.17 (0.07-0.42)	<0.001	--	
BMI category§					
	Normal/underweight	Ref		Ref	
	Overweight/obese	1.38 (1.08-1.76)	0.011	1.32 (1.03-1.70)	0.027
Elevated triglycerides‖ during follow-up					
	Never	Ref		Ref	
	Ever	0.87 (0.67-1.13)	0.28	0.89 (0.67-1.18)	0.40
	Missing	0.29 (0.14-0.61)	0.001	0.55 (0.23-1.29)	0.17

Data are from individuals with HBV and HIV enrolled in the ATHENA cohort, including information from cohort inclusion until the last clinical visit from 2000-2022. In this analysis, only those with positive hepatitis B surface antigen at their last follow-up visit were included. This represents 1,243 individuals, of whom 209 were tested for HDV. All characteristics refer to the date of last clinical visit unless otherwise specified.

†Individuals who never had a given marker measured were considered belonging to the "never" group.

‡Defined as an APRI score >2.0 or liver stiffness measurement ≥9.5 kPa.

§Defined by World Health Organization categories: normal/underweight, <25 kg/m^2; overweight, 25-30 kg/m^2; obese, >30 kg/m^2.

‖Defined as serum triacylglycerol >1.7 mmol/L.

€All parameter estimates were adjusted by clinical site.

¥Selection of the variables included in the multivariable model are indicated in Table 2.

--, category level was excluded from analysis.

Abbreviations: ALT, alanine aminotransferase; APRI, aspartate aminotransferase to platelet ratio index; aRR, adjusted relative risk; ART, antiretroviral therapy; BMI, body mass index; CI, confidence interval; HBV, hepatitis B virus; HDV, hepatitis D virus; RR, relative risk; ULN, upper limit of normal.
